# Platelet transfusion enhances pro‐aggregatory status shortly after coronary artery bypass grafting (CABG while modulating platelet pro‐inflammatory state 1‐week post‐surgery

**DOI:** 10.1111/jcmm.18573

**Published:** 2024-08-09

**Authors:** Javad Ahmadi, Ehteramolsadat Hosseini, Faranak Kargar, Mahtab Maghsudlu, Mehran Ghasemzadeh

**Affiliations:** ^1^ Blood Transfusion Research Center High Institute for Research and Education in Transfusion Medicine Tehran Iran; ^2^ Torbat Heydariyeh University of Medical Sciences Torbat Heydariyeh Iran; ^3^ Rajaie Cardiovascular Medical and Research Center Iran University of Medical Science Tehran Iran

**Keywords:** CD40 ligand, coronary artery bypass grafting (CABG), coronary artery disease (CAD), inflammation, PAC‐1 binding, platelet storage lesion, P‐selectin, thrombosis, transforming growth factor‐β1 (TGF‐β1), transfusion

## Abstract

During coronary artery bypass grafting (CABG), the surgical procedure, particularly the manipulation of the major arteries of the heart, induces a significant inflammatory state that may compromise platelet function to the extent that platelet transfusion is required. Given stored platelets as a major source of biological mediators, this study investigates the effects of platelet transfusion on the major pro‐aggregatory, pro‐inflammatory and immunomodulatory markers of platelets. Platelets from 20 patients, 10 who received platelet transfusion and 10 without, were subjected to flow cytometery where P‐selectin and CD40 ligand (CD40L) expressions and PAC‐1 binding (activation‐specific anti GPIIb/GPIIIa antibody) analysed at five‐time points of 24 h before surgery, immediately, 2 h, 24 h and 1 week after surgery. Analysis of intra‐platelet transforming growth factor‐beta‐1 (TGF‐β1) was also conducted using western blotting. Patients with platelet transfusion showed increased levels of P‐selectin, CD40L and intra‐platelet TGF‐β1 2‐h after surgery compared to those without transfusion (*p* < 0.05). PAC‐1 binding was increased 24 h after surgery in transfused patients (*p* < 0.05). Given the significant post‐transfusion elevation of platelet TGF‐β1, P‐sel/CD40L reduction in transfused patients a week after was of much interest. This study showed for the first time the significant effects of platelet transfusion on the pro‐inflammatory, pro‐aggeregatory and immunomodulatory state of platelets in CABG patients, which manifested with immediate, midterm and delayed consequences. While the increased pro‐inflammatory conditions manifested as an immediate effect of platelet transfusion, the pro‐aggregatory circumstances emerged 24 h post‐transfusion. A week after surgery, attenuations of pro‐inflammatory markers of platelets in transfused patients were shown, which might be due to the immunomodulatory effects of TGF‐β1.

## 1 INTRODUCTION

Coronary artery bypass grafting (CABG) is a surgical intervention that is often used to treat patients with coronary artery disease (CAD).^1^ Although the overall results of CABG surgery have improved significantly, the procedure can still be associated with some risk of mortality and morbidity.^2^, ^3^, ^4^ While the most critical complications of CABG can be caused by problems in the surgery itself, including aortic manipulation, improper clamping or unclamping leading to thromboembolism, or graft occlusion due to surgical errors, post‐surgery maintenance of patients is also considered  as a critical point that is relevant to the either short‐ or long‐term complications.^5^, ^6^ This situation is also more complicated in patients with underlying diseases that affect platelet function, which can require additional therapeutic interventions, including platelet transfusions during surgery.^6^ Studies indicate that CAD patients undergoing CABG are the second most consumers of platelet products after those with blood malignancies, and basically platelet transfusion is one of the most common therapeutic interventions for them, both prophylactically or therapeutically.^7^, ^8^, ^9^, ^10^, ^11^, ^12^

As a highly invasive intervention, in addition to sternal splitting and rib expansion before cardiopulmonary bypass (CPB), during the CABG procedure itself, surgical manipulation of major cardiac vessels also imposes significant inflammatory conditions on patients.^13^, ^14^ This intense inflammatory state described by the elevation of the vast arrays of inflammatory cytokines, including IL‐1, ‐6, ‐8 and TNF‐α, can also affect platelet function post‐surgery.^15^, ^16^

So far, several lines of evidence have shown that platelets from patients undergoing CABG have a pro‐inflammatory phenotype.^17^, ^18^, ^19^ On the other hand, significant platelet dysfunction in some patients may cause postoperative bleeding, which inevitably makes them candidates for platelet transfusion.^20^, ^21^, ^22^ However, regardless of the different techniques used for the preparation or storage of banked platelets, these products may be partially activated by the well‐known process of platelet storage lesion (PSL),^23^, ^24^ which can lead to imposing some unwanted effects on patients after platelet transfusion.

It has been shown that the storage of therapeutic platelets in vitro is associated with some signs of platelet pre‐activation, including enhanced pro‐aggregatory behaviour and elevated levels of activation markers of platelets, including P‐selectin, CD40 ligand (CD40L) and active transforming growth factor‐beta 1 (TGF‐β1).^25^ Although in the best‐case scenario, the low levels of pre‐activation may not significantly affect the post‐transfusion function of platelets, in post‐CABG conditions, the exposure of these pre‐activated platelets to an overt inflammatory milieu may enhance platelet functional activity further, significantly increasing the pro‐inflammatory function of transfused platelets.^18^, ^26^ To better address the importance of such changes after platelet transfusion, specific tracking experiments (using genetically integrated markers or platelet labelling) in preclinical murine models could be good approaches to monitors the functional efficacy and fate of stored platelet products after transfusion.^27^, ^28^, ^29^

The importance of this issue arises from the fact that during surgery, the primary attention of the surgical team is expertly focused on the control and management of the patient's homeostasis on both sides of thrombosis and bleeding,^1^ but what is sometimes overlooked is the bilateral interaction of the inflammatory state caused by platelet transfusion with the hemostatic conditions of the patients.^23^ In this regard, paying full attention to both changes in the pro‐aggregatory and pro‐inflammatory profiles of platelets before and after platelet transfusion will be very important because although hemostatic complications can be medically prevented during surgery and shortly after, the inflammatory state of transfused platelets left without basic control may cause some short‐term and long‐term complications by inducing thrombo‐inflammation.^11^ This means that, while the need for platelet transfusion per se represents a more vulnerable state of patients, given the pro‐inflammatory condition that the transfused platelets themselves may mediate, this therapeutic intervention may be a case of risk–benefit judgement that requires further investigation. In other words, regardless of the importance that platelet transfusion has in supporting proper haemostasis in high‐risk patients, it sounds reasonable to investigate whether transfused platelets, which are a major source of pro‐inflammatory and immunomodulatory mediators, can influence the inflammatory status of CABG patients or not. In other words, whereas in the short‐term, the exposure of freshly transfused platelets to the post‐surgery inflammatory milieu can be associated with unwanted activation of platelets, which may overshadow the beneficial effect of platelet transfusion, the immunomodulatory effects of these platelets, which are rich in TGF‐β1, unlike the exhausted platelets of patients, can moderate the post‐CABG inflammatory state after a longer time, which may indirectly affect platelet function.

Therefore, the study presented here for the first time aimed to investigate the most important pro‐aggregatory, pro‐inflammatory and immunomodulatory markers of platelets, including PAC‐1 binding, P‐selectin, CD40 ligand and intra‐platelet content of TGF‐β1, after GABG in patients with and without platelet transfusion, so that it provides a comprehensive view of the effect of platelet transfusion on the patient's platelet functional activity status by providing a comparative analysis.

## 2 MATERIALS AND METHODS

### 2.1 Reagents and chemicals

The fluorescein isothiocyanat isothiocyanate (FITC)‐ or phycoerythrin (PE)‐conjugated mouse IgG1 isotype controls were provided by Miltenyi Biotec (Germany). The human monoclonal antibodies CD41 (FITC/PE‐conjugated), CD62P (PE‐conjugated), CD40L (FITC‐conjugated) and PAC‐1 (FITC‐coupled activation‐specific anti‐GPIIb/IIIa antibody) were supplied by BD Pharmingen. The anti‐human TGF‐β1 mouse monoclonal antibody and anti‐β‐actin were acquired from Santa Cruz Biotechnology (USA). The HRP‐conjugated antibody was also provided by Santa Cruz Biotechnology (USA). The remaining chemicals and reagents were obtained from Sigma Aldrich. The Sysmex K‐x21 instrument quantified mean platelet volume (MPV), platelet and white blood cell (WBC) count.

### 2.2 Patients

The study comprised a total of twenty patients, consisting of fifteen men and five females, who had stable coronary artery disease (CAD) and were eligible for elective on‐pump coronary artery bypass grafting (CABG) at the Rajaie Cardiovascular Medical and Research Center in Tehran, Iran. This study comprised a cohort of individuals diagnosed with advanced CAD who had notable similarities in their demographic and clinical characteristics. All study participants showed a stenosis of 75%–95%, which met the criteria set for the classification of “significant CAD” (stenosis greater than 50%). In this study, 17 out of 20 people had three‐vessel (3 V) involvement. The left ventricular ejection fraction (LVEF) showed a somewhat uniform pattern among the patients, as evidenced by the fact that 16 individuals had an LVEF above 50%. Each patient adhered to a daily dosage of 80 mg of aspirin until the day preceding the surgical procedure. Before CABG, patients were subjected to a series of screening tests, including complete blood count (CBC), liver functional test (LFT), kidney and thyroid function tests, erythrocyte sedimentation rate (ESR) and pulmonary function test (PFT), to evaluate the potential risk of developing lung disorders. In addition, individuals aged 65 and above were subjected to an echocardiogram to assess cardiac ejection fraction (EF) and carotid Doppler testing to evaluate the presence of obstructive lesions in the carotid arteries in the head and neck region. Participants who had a cancer diagnosis, chronic liver illness, renal insufficiency, connective tissue disease, or were currently using anti‐inflammatory, clopidogrel, or anticoagulant drugs were excluded from the study. Table 1 provides a summary of the demographic and clinical characteristics of the patients. Written informed consent was obtained from all participants. The research was conducted in accordance with the principles embodied in the Declaration of Helsinki and received approval from a regional ethics board. This study was approved in 2020 by the Ethics Committee of the High Institute for Research and Education in Transfusion Medicine, Tehran, Iran (Ethics approval number: IR.TMI.REC.1398.012).

**TABLE 1:** Demographic and clinical characteristics of patients with and without platelet transfusion.

Characteristics	Patients with Plt (*n* = 10)	Patients without Plt (*n* = 10)
Age (mean ± SD)	62.4 ± 5.5	60.6 ± 6.9
Sex		
Male (%)	7 (70)	8 (80)
Female (%)	3 (30)	2 (20)
Diagnosis		
2VD (%)	1 (10)	2 (20)
3VD (%)	9 (90)	8 (80)
Stenosis		
>65% (%)	10 (100)	10 (100)
LVEF		
≤40% (%)	1 (10)	0 (0)
41%–49% (%)	2 (20)	1 (10)
≥50% (%)	7 (30)	9 (90)
CBC parameters		
WBC count (×10^3^/μL; mean ± SD)	7.8 ± 1.6	8.1 ± 1.3
Neutrophil (%)	62.9 ± 9.2	62.4 ± 9.1
Lymphocyte (%)	29.1 ± 7.7	29.6 ± 7.8
NLR (%)	2.16 ± 0.63	2.1 ± 0.77
Platelet count (×10^3^/μL; mean ± SD)	224.6 ± 38.56	250.4 ± 55.92
Biochemistry parameter		
Total cholesterol (mg/dL)	138.6 ± 27.54	135.4 ± 27
HDL (mg/dL)	37.23 ± 8.31	35.26 ± 8.46
LDL (mg/dL)	79.1 ± 26.71	78.73 ± 29.2
BUN (mg/dL)	14 ± 2.67	13.63 ± 2.15
Creatinine (mg/dL)	1 ± 0.17	0.96 ± 0.15
Inflammatory markers		
CRP (mg/L)	9.1 ± 7.7	8.97 ± 1.58
ESR (mm/h)	25.77 ± 14.12	23.86 ± 3.06
The number of transfused units	2	_
The lifespan of transfused units (days)	3	_

Abbreviations: LVEF, left ventricular ejection fraction; NLR, neutrophil to lymphocyte ratio; VD, vessel disease.

### 2.3 Surgery procedure

Uniform administration of anaesthesia was applied to all patients in both induction and maintenance phases, while surgical procedures were performed through median sternotomy. Cardiopulmonary bypass (CPB) was performed using a heart–lung machine prepared with a solution consisting of 1000 mL of Ringer's acetate, 200 mL of mannitol and a dosage of heparin equivalent to 100 units per kilogram (or 1 mg/kg). A roller pump was employed for all patients. Before the surgical procedure, every patient was administered a heparin dosage of 350 units per kilogram (or 3.5 mg/kg). This was done to regulate the activated clotting time (ACT) within 400–480 s. Heparin dosages were augmented during the bypass process to sustain a therapeutic‐activated clotting time that exceeded 480 seconds. After the surgical procedure, the administration of protamine sulphate was employed to counteract the anticoagulant effects of heparin. The protamine administration occurred at a dosage range of 1–1.3 mg per 100 units of heparin.

### 2.4 Sample collection

For this study, peripheral blood samples from all patients were obtained using sodium citrate 3.2% anticoagulant at five specific time intervals: 24 h before the surgery, immediately after the surgery, 2 h after the surgery, 24 h after the surgery and 1 week after the surgery. Following the surgical procedure, the patient was disconnected from the pump. Subsequently, 15 min after the protamine injection to neutralise the heparin, the second blood sample was collected (immediately after the surgery). After the blood extraction, the surgeon examined the surgical incision site for the level of oozing. Therefore, based on the observation of excessive oozing, the surgeon decided to transfuse platelets into some patients who subsequently formed the second study group, which included patients with platelet transfusion. All patients were transfused with 2 units of concentrated platelets with a lifespan less than 3 days via central venous catheter. Patients were transferred to the Intensive Care Unit (ICU), and third/fourth blood samples were collected 2 and 24 h after surgery, respectively. Finally, the patients were transferred to the surgery department, where the fifth blood sample was taken 1 week after the surgery. All samples were transferred to the laboratory immediately after collection under temperature and mechanical controlled conditions for platelet‐rich plasma (PRP) preparation.

### 2.5 Platelet‐rich plasma preparation

Blood samples obtained from patients at various intervals were centrifugated with a force of 150 g for 10 min at room temperature. This resulted in the generation of platelet‐rich plasma (PRP). The platelet count in each PRP preparation was determined using a Sysmex Kx‐21 instrument. To ensure a rest period, all PRP preparations were subjected to incubation at 37°C for a minimum of 30 min before conducting flow cytometry analysis on P‐selectin, CD40L and PAC1. The platelet counts were adjusted to a concentration of 2 × 10^7^ cells/mL using Tyrode buffer to perform flow cytometry analysis. For western blot analysis, washed platelets with a count of 1 × 10^8^ cells/ml were re‐suspended in Tyrode buffer.

### 2.6 Flow cytometry

Platelets at a concentration of 2 × 10^7^/ml using Tyrode buffer (10 mM HEPES, 12 mM NaHCO_3_, 137 mM NaCl, 2.7 mM KCl, 5 mM glucose, 1 mM CaCl_2_; pH 7.2–7.4) were incubated in the presence of antibodies (at 2–5 μg/mL dilution) targeting P‐selectin (CD62P), CD40L, or PAC‐1. The manufacturer's instructions specified the amount of each antibody used. After the incubation period, the cells were then fixed by adding an equal volume of 1% paraformaldehyde solution in phosphate‐buffered saline (PBS, PH = 7.2–7.4). Subsequently, the fixed cells were analysed using a flow cytometer (CyFlow Space, Partec GmbH and Germany) to get a total count of 20,000 platelet events. The flow cytometer was configured to optimise the acquisition of platelets by adjusting the forward and side scatter channels, as well as the fluorescence channels FL1/FL2. Logarithmic signal amplification was employed in all four detectors. The gate was strategically placed around a population of platelets that exhibited intact forward and side scatter properties, and the presence of platelets expressing CD41 further validated this. The percentages of positive platelets expressing P‐selectin and CD40L above the background (isotype control) were calculated, where the PAC‐1 binding was evaluated with mean fluorescence intensity (MFI). The data was analysed using FLOWJO software (Tree Star Inc., OR, USA).

### 2.7 Western blotting

To evaluate the total TGF‐β1 content of the platelet, the platelet concentration of each sample obtained from the PRP was adjusted to a count of 1 × 10^8^/ml using Tyrode buffer (10 mM HEPES, 12 mM NaHCO3, 137 mM NaCl, 2.7 mM KCl, 5 mM glucose, 1 mM CaCl2; pH 7.2–7.4). 1 mL of each sample was then subjected to a soft spin centrifugation (2000 *g*) for 5 min at 4°C. The pellets of platelets were subsequently lysed on ice using Lysis buffer containing 100 mM EDTA and Triton 10× in PBS supplemented with a protease inhibitors cocktail. The lysates were then subjected to a second centrifugation step at 7000 *g* for 10 min at 4°C, and the supernatants were collected for further analysis by western blotting. For this purpose, a volume of 25 μL from each supernatant was first subjected to sodium dodecyl‐sulphate polyacrylamide gel electrophoresis (SDS‐PAGE), where the separated protein bands were then transferred to the polyvinylidene fluoride (PVDF) membrane. After initial blocking and washing steps, the corresponding TGF‐β1 band was sequentially immunoblotted in the presence of a TGF‐β1‐specific monoclonal antibody, followed by the incubation with a secondary antibody coupled to HRP. Finally, the bands were visualised and analysed by the ChemiDoc XRS+ equipment and Imaging Lab software (Bio‐Rad Laboratories, Inc., USA). In parallel, as a housekeeping protein, β actin re‐staining was also performed for every experiment. For comparison, the intensity ratio of each sample to its matching β actin intensity was calculated to rule out the potential of inconsistent loading of the total protein extract^30^ (Figure S1).

#### 2.7.1 Statistical analysis

The data was analysed using GraphPad Prism software (GraphPad Prism Software, Inc., San Diego, CA). The mean and standard deviation (SD) were calculated. For comparison of the parameters on different days in each group, data were analysed by the ANOVA and Kruskal–Wallis test with Dunn's multiple comparison tests. The t‐test and Mann–Whitney U test were also applied to compare parameters between the two groups. Significance was attributed to *p*‐values that were less than 0.05.

## 3 RESULTS

### 3.1 Comparison of platelet count, White blood cell, neutrophil‐to‐lymphocyte ratio and mean platelet volume in patients with and without platelet transfusion

**FIGURE 1:**
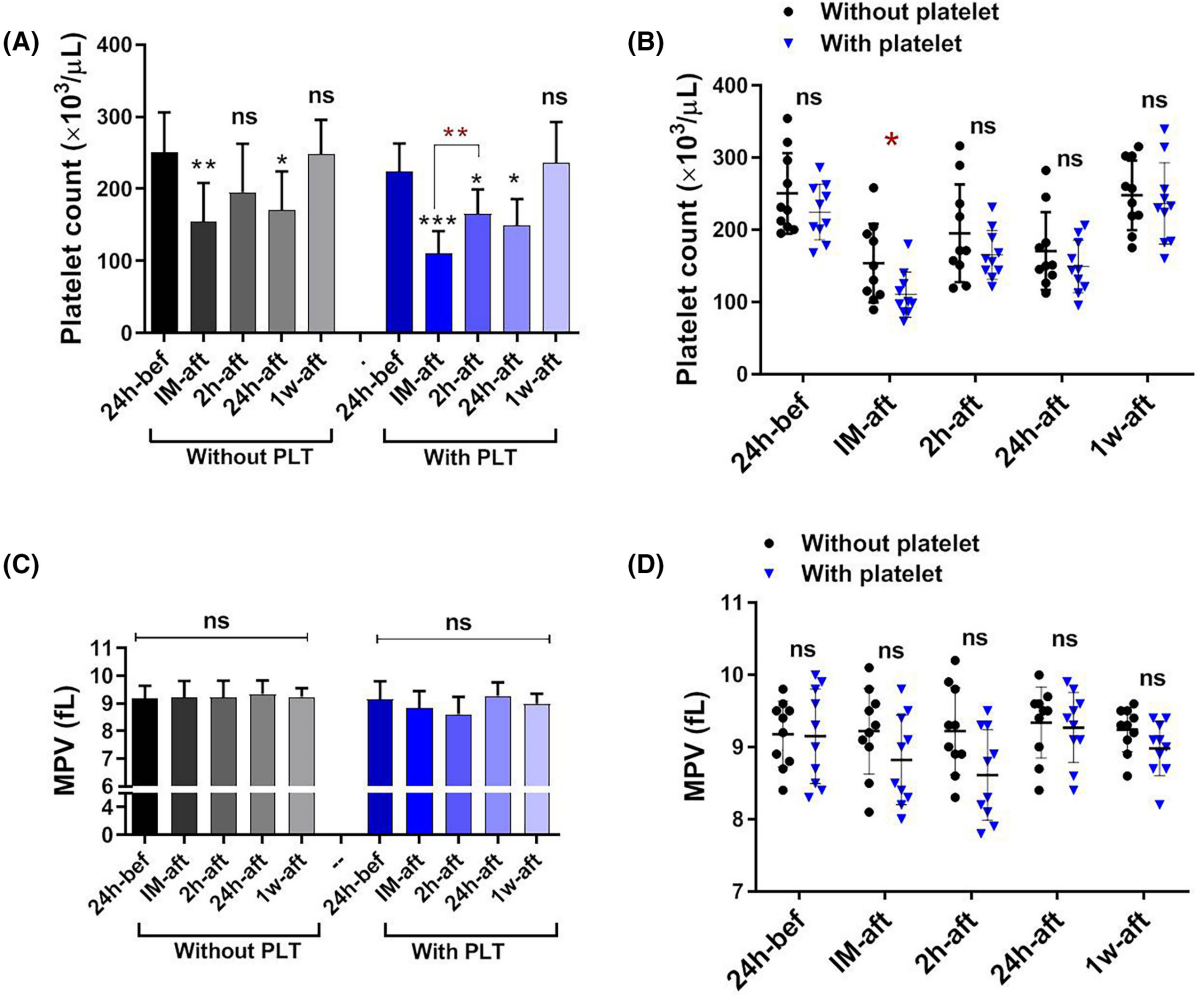
The platelet count and MPV changes in patients with and without platelet transfusion. Both patient groups with and without platelet transfusion observed a statistically significant platelet count reduction (*p* < 0.05) immediately following the surgery (A). For easier comparison and interpretation of the results between the two groups, the results were compared side by side; Notably, platelet count reduction was more significantly in patients with platelet transfusions (*p* < 0.05) (B). A comparison of MPV revealed no significant difference between the two groups of patients (C, D). bef; before, IM‐aft; immediately after, 2 h‐aft; 2 h after, 24 h‐aft; 24 h after, 1w‐aft; 1 week after, ns; not significant. For comparison of the parameters on different days in each group, data were analysed by the Kruskal–Wallis test with Dunn's multiple comparison tests. The Mann–Whitney *U* test was also applied to compare parameters between the two groups. (*p < 0.05, **p < 0.01, ***p < 0.001). (*n* = 20).

To ascertain the temporal trends in platelet and WBC count, as well as the neutrophil‐to‐lymphocyte ratio (NLR) and mean platelet volume (MPV) among patients who received platelet concentrates and those who did not, we examined and compared the respective values at various time intervals (24‐h pre‐surgery, immediately, 2‐h, 24‐h and 1‐week post‐surgery) within two distinct patient cohorts. As depicted in Figure 1A, both patient groups observed a statistically significant platelet count reduction (*p* < 0.01; *p* < 0.001) immediately after surgery, whereas the comparison of the two groups before platelet transfusion showed that the number of platelets in the group of patients who required therapeutic platelets was significantly lower than the other group (*p* < 0.05; Figure 1B). Therefore, in addition to the observation of excessive oozing, this issue can be the reason that justifies the increased need for platelet transfusions in a group of patients. After the decrease, a transient rise in platelet count was observed 2‐h post‐surgery, which again partially declined 24 h later. Subsequently, the platelet count gradually reverted to baseline within 1 week following the surgery.

**FIGURE 2:**
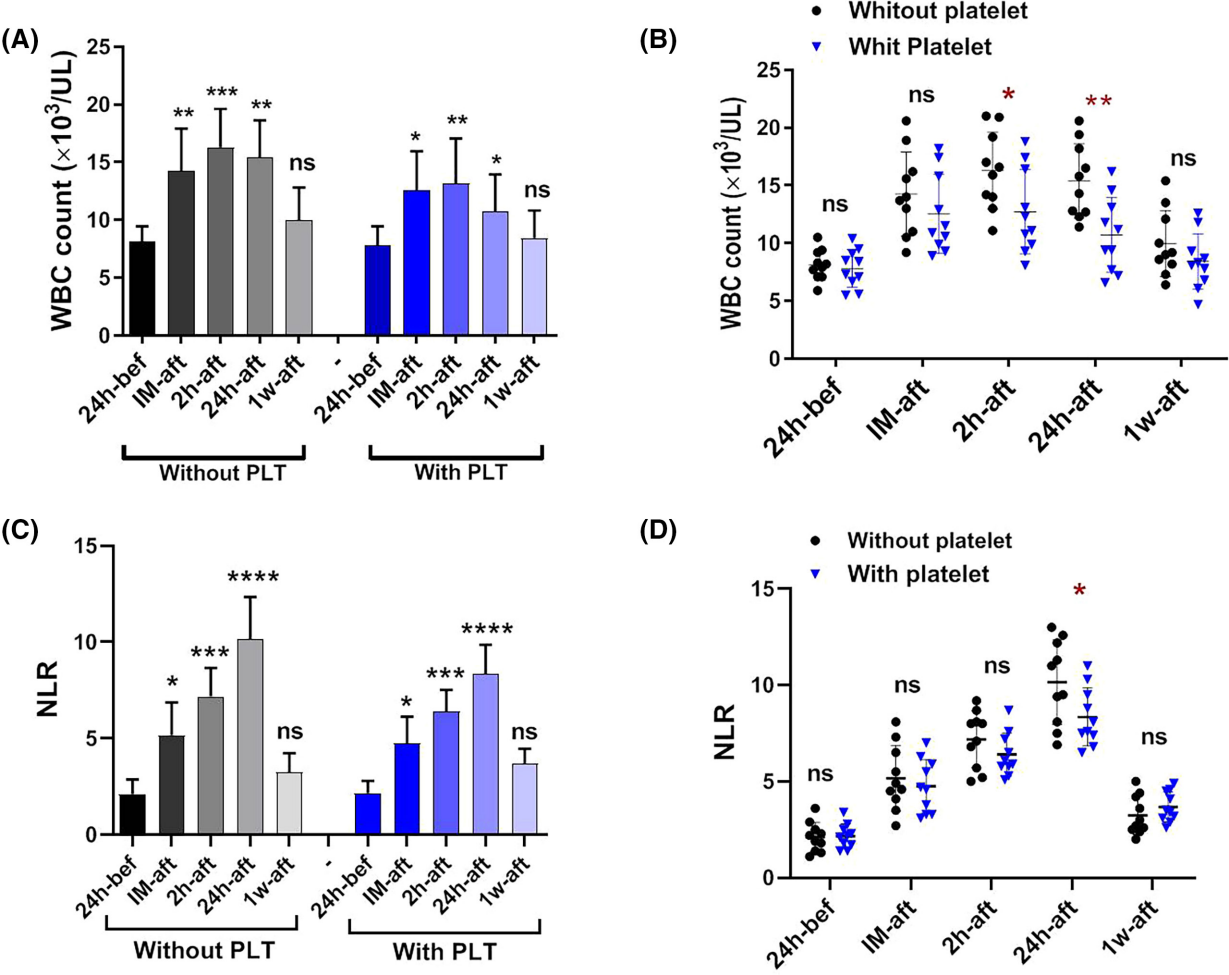
The WBC count and NLR changes in patients with and without platelet transfusion. The WBC count in both patient groups with and without platelet transfusion exhibited a notable increase (*p* < 0.05) immediately, 2 h and 24 h post‐surgery, compared to the pre‐surgery values (A). For easier comparison and interpretation of the results between the two groups, the results were compared side by side. The WBC count was seen significantly (*p* < 0.05) lower in the group of patients who received platelets over the time intervals of 2 and 24 h after the surgery (B). The NLR exhibited a statistically significant rise post‐surgery (*p* < 0.05) (C). When the results of two groups were compared side by side, NLR exhibited a significant decrease (*p* < 0.05) in patients who underwent platelet transfusion compared to those who did not receive platelets, specifically during 24 h following surgery (D). bef; before, IM‐aft; immediately after, 2 h‐aft; 2 h after, 24 h‐aft; 24 h after, 1w‐aft; 1 week after, ns; not significant. For comparison of the parameters on different days in each group, data were analysed by the Kruskal–Wallis test with Dunn's multiple comparison tests. The Mann–Whitney *U* test was also applied to compare parameters between the two groups (*p < 0.05, **p < 0.01, ***p < 0.001, ****p < 0.0001). (*n* = 20).

The WBC count in both patient groups exhibited a notable rise immediately, 2 h and 24 h post‐surgery, compared to the pre‐surgery measurements. However, the WBC count reverted to its initial levels a week after the surgical procedure (Figure 2A). Nevertheless, comparing WBC counts between patients who did not receive platelets and those who did showed lower increases in WBC counts in the group of patients who received platelets. This significant difference was observed over the time intervals of 2 h (*p* < 0.05) and 24 h after the surgery (Figure 2B).

The NLR exhibited a statistically significant rise post‐surgery (Figure 2C). The NLR peaked 24 h after the surgery and reverted to preoperative baseline values within 1 week. Notably, similar to the number of WBCs, the level of NLR as an indicator of inflammation exhibited a statistically significant decrease in patients who underwent platelet transfusion compared to those who did not receive platelets, specifically during 24 h (*p* < 0.05) after surgery (Figure 2D).

The analysis and comparison of MPV revealed no statistically significant difference between the two groups of patients (Figure 1C,D).

### 3.2 Comparison of P‐selectin expression patterns in patients with and without platelet transfusion

**FIGURE 3:**
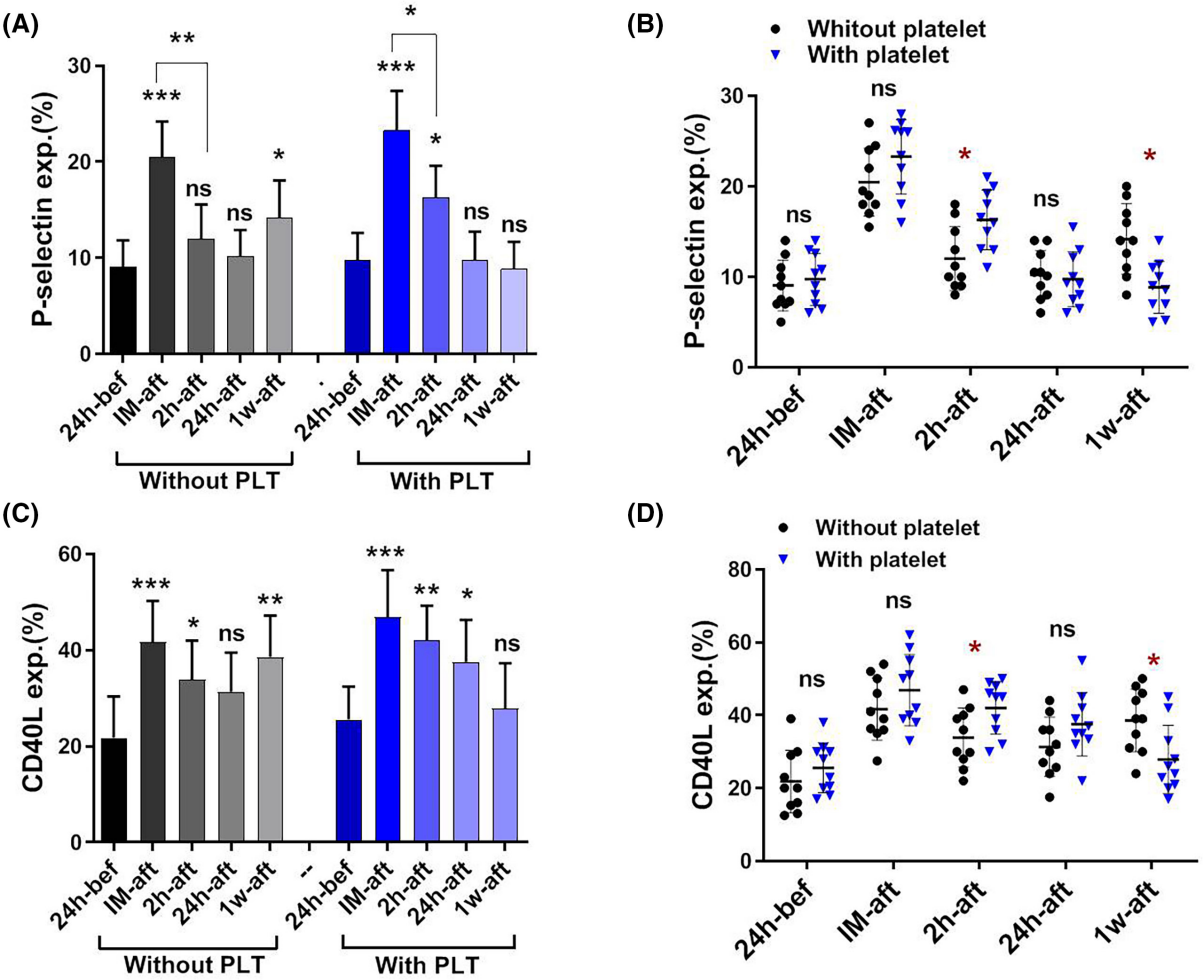
The P‐selectin and CD40L expression patterns in patients with and without platelets transfusion. A significant increase (*p* < 0.001) in the expression of P‐selectin and CD40L was observed immediately following surgery and followed by a decrease to 24 h in patients with and without platelet transfusion. Significantly, among patients who did not receive platelet transfusion, there was a notable increase (*p* < 0.05) in the expression of P‐selectin and CD40L 1 week post‐surgery compared to the baseline values observed before surgery (A, C). When the results of two groups were compared side by side, in patients who receive platelets, the levels of P‐selectin and CD40L expression 2‐h post‐surgery were significantly elevated compared to patients who did not receive platelets, and also, these patients did not exhibit an increase in P‐selectin and CD40L expression levels 1 week post‐surgery (B, D). bef; before, IM‐aft; immediately after, 2 h‐aft; 2 h after, 24 h‐aft; 24 h after, 1w‐aft; 1 week after, ns; not significant. For comparison of the parameters on different days in each group, data were analysed by the Kruskal–Wallis test with Dunn's multiple comparison tests. The Mann–Whitney *U* test was also applied to compare parameters between the two groups (*p < 0.05, **p < 0.01, ***p < 0.001). (*n* = 20).

According to the data presented in Figure 3A, there was a statistically significant increase (*p* < 0.001) in the expression of P‐selectin immediately following surgery in patients who did not receive platelets, compared to the expression levels observed 24 h before the surgery. The observed values after 2‐ and 24‐h post‐surgery exhibited statistically significant decreases compared to the immediate post‐surgery period. Significantly, among patients who did not receive platelet transfusion, there was a notable increase (*p* < 0.05) in the expression of P‐selectin 1‐week post‐surgery compared to the baseline values observed before surgery.

Patients who received platelet transfusion had a similar pattern, but with the notable exception that the levels of P‐selectin expression 2 h post‐surgery were significantly elevated compared to patients who did not receive platelets (*p* < 0.05). Furthermore, it was noted that patients who received platelet transfusion did not exhibit an increase in P‐selectin expression levels 1‐week post‐surgery, in contrast to patients who did not receive platelets (Figure 3B).

### 3.3 Comparison of CD40L expression patterns in patients with and without platelet transfusion

The expression of CD40L in patients who did not receive platelets had a similar trend to that of P‐selectin. In particular, the expression of CD40L was significantly increased from baseline immediately after surgery. Subsequently, a gradual decrease was observed until 24 h after surgery. However, 1 week after surgery, a significant increase in the expression of this marker was observed, which brought it back to higher levels than before surgery (*p* < 0.01; Figure 3C).

The pattern above was observed in individuals who received platelet transfusion, as depicted in Figure 3C. However, it is worth noting that the expression level of CD40L was considerably elevated 2‐h post‐surgery in patients who received platelets compared to those who did not (*p* < 0.05). Furthermore, it was observed that patients who had platelet transfusion exhibited a decreased expression level of CD40L 1‐week post‐surgery, in contrast to those who did not undergo platelet transfusion (*p* < 0.05; Figure 3D).

### 3.4 Comparison of total transforming growth factor‐β1 (TGF‐β1) content of platelets in patients with and without platelet transfusion

**FIGURE 4:**
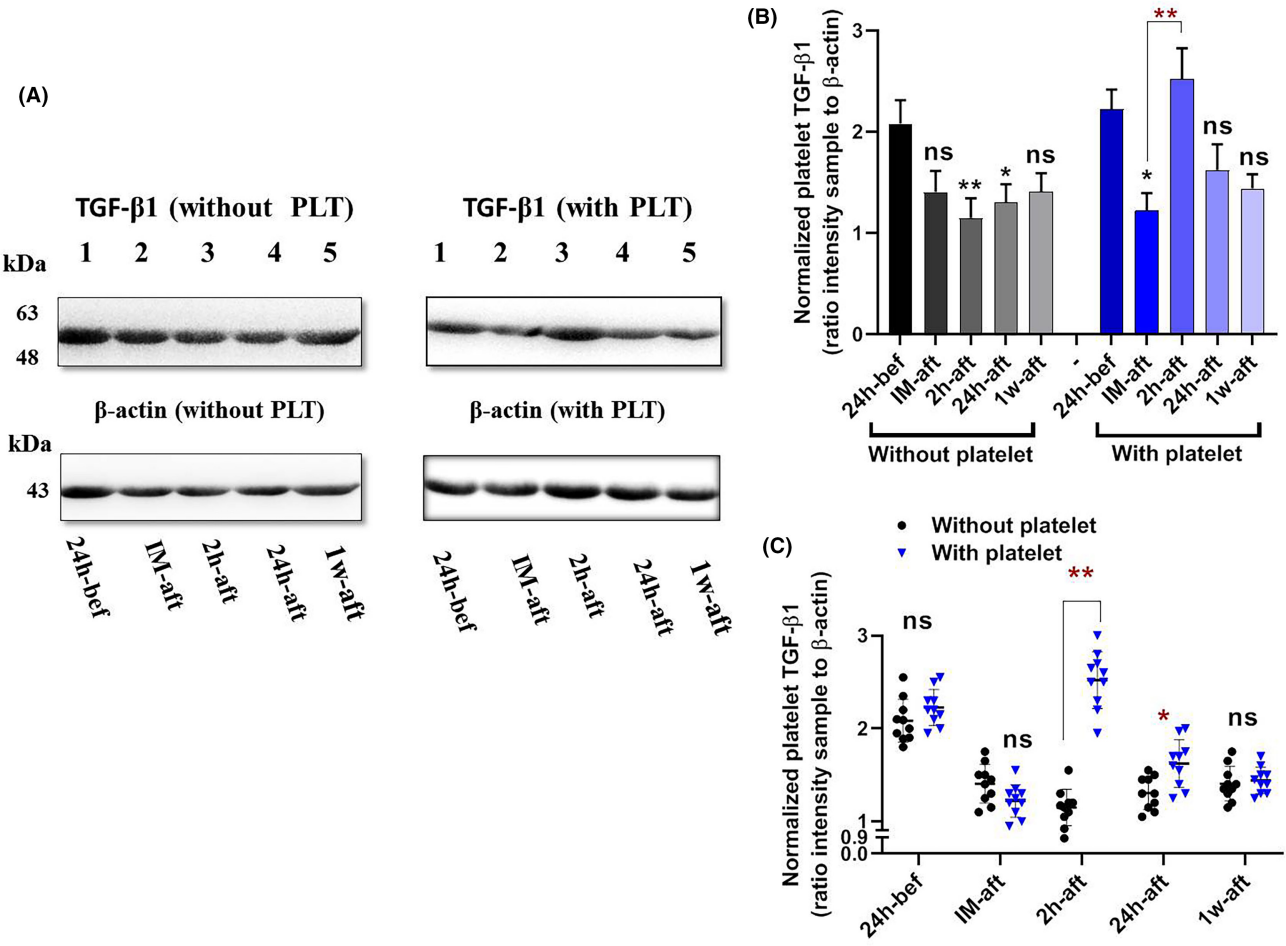
The intra‐platelet TGF‐β1 content in patients with and without platelet transfusion. Western blot analysis and parallel‐actin staining were conducted to assess intra‐platelet TGF‐β1 content in patients with and without platelet transfusion (A). A significant decrease (*p* < 0.001) was detected in TGF‐β1 levels 2‐h post‐surgery in patients who did not receive platelets. In return, A significant increase (*p* < 0.001) was detected in TGF‐β1 content 2‐h post‐surgery in patients who received platelet (B). When the results of two groups were compared side by side, there was a significant difference in the content of TGF‐β1 between the two groups at the 2‐ and 24‐h post‐surgery time points (C). bef; before, IM‐aft; immediately after, 2 h‐aft; 2 h after, 24 h‐aft; 24 h after, 1w‐aft; 1 week after, ns; not significant. For comparison of the parameters on different days in each group, data were analysed by the Kruskal–Wallis test with Dunn's multiple comparison tests. The Mann–Whitney U test was also applied to compare parameters between the two groups (*p < 0.05, **p < 0.01). (*n* = 20).

The samples were subjected to western blot analysis at different time courses to detect the total TGF‐β1 content of platelets (intra‐platelet TGF‐β1) in patients with and without platelet transfusion. Parallel‐actin staining was also applied to each blot, and the ratio of the intensity of each sample to the corresponding actin intensity was calculated (Figure 4A). The analysis of the TGF‐β1 to β‐actin ratio as an internal control revealed a significant decrease (*p* < 0.01) in TGF‐β1 levels 2‐h post‐surgery in patients who did not receive platelets. Subsequently, TGF‐β1 levels exhibited an upward trend, gradually increasing until 1 week following the surgery. In return, the analysis of the TGF‐β1 to β‐actin ratio in individuals who underwent platelet transfusion revealed that following the transfusion (specifically, 2‐h post‐surgery), the concentration of TGF‐β1 was significantly increased compared to that of immediately after surgery (Figure 4B). One possible explanation is the high level of total TGF‐β‐1 content in the transfused platelet products, which increases the post‐infusion level of intraplatelet TGF‐β1 in the circulation, where it now contains fresh platelets alongside the exhausted platelets of patients which had previously lost their TGF‐β1 content. In this regard, a significant difference in TGF‐β1 content was observed between the two groups at 2 (*p* < 0.01) and 24 h (*p* < 0.05) after surgery (Figure 4C).

### 3.5 Comparison of platelet protease‐activated receptor 1 (PAC‐1) binding capacity in patients with and without platelet transfusion

**FIGURE 5:**
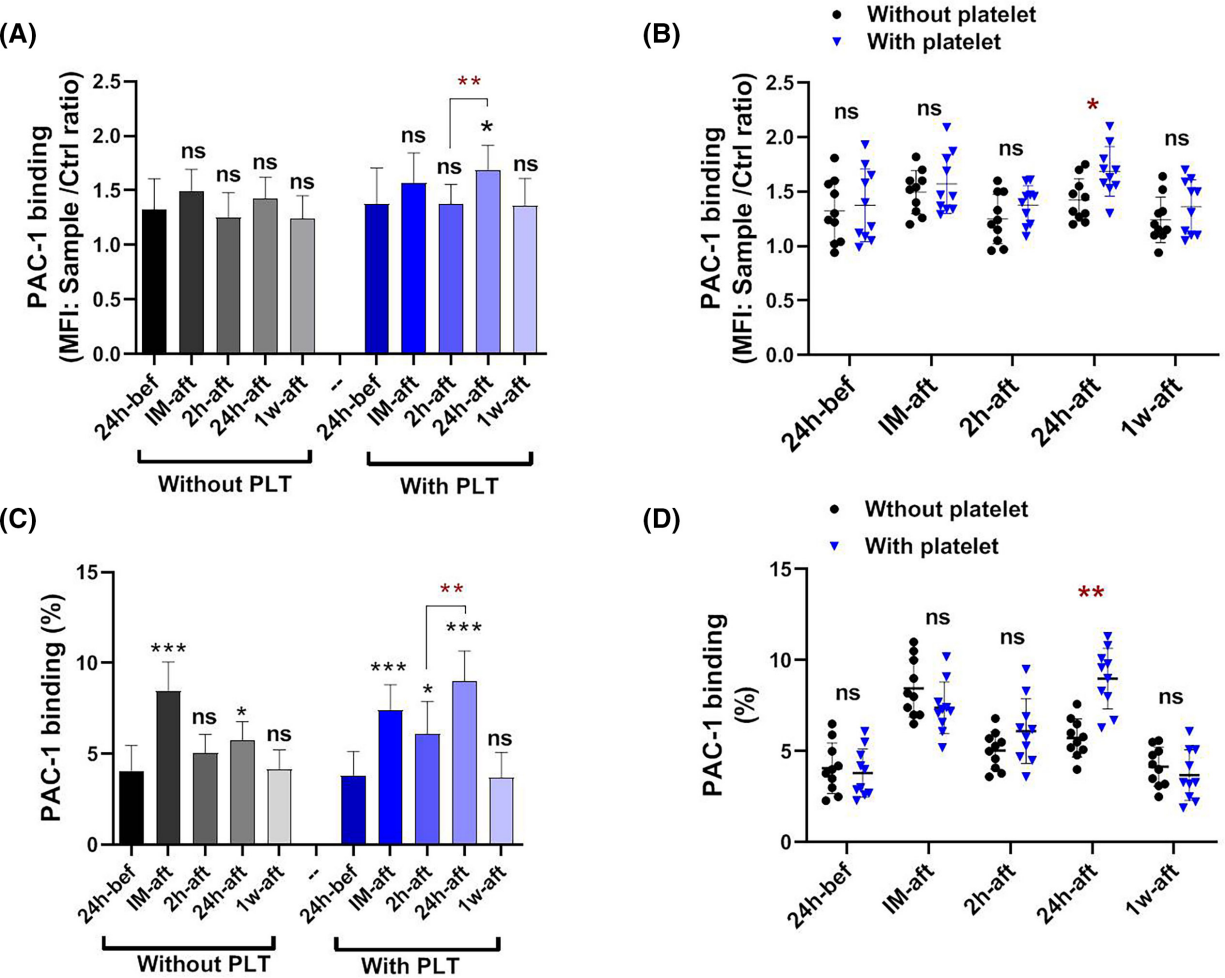
The PAC‐1 binding capacity in patients with and without platelet transfusion. PAC‐1 binding was analysed in both groups of patients based on MFI. Results did not show significant changes in PAC‐1 binding (MFI) in patients without platelet transfusion; however, a significant increase was demonstrated in patients with platelet transfusion 24 h after surgery (A). When the results of two groups were compared side by side, a significant difference in PAC‐1 binding was seen in patients receiving platelets compared to the other group 24 h after the surgery (B). bef; before, IM‐aft; immediately after, 2 h‐aft; 2 h after, 24 h‐aft; 24 h after, 1w‐aft; 1 week after, ns; not significant. For comparison of the parameters on different days in each group, data were analysed by the Kruskal–Wallis test with Dunn's multiple comparison tests. The Mann–Whitney U test was also applied to compare parameters between the two groups (*p < 0.05, ** *p* < 0.01, *** *p* < 0.001). (*n* = 20).

As presented in Figure 5A, while it did not show significant changes in PAC‐1 binding (MFI) in patients without platelet transfusion, a considerable increase was demonstrated in patients with platelet transfusion 24 h after surgery. A significant difference in PAC‐1 binding was seen in patients receiving platelets compared to the other group 24 h after the surgery (Figure 5B).

## 4 DISCUSSION

As results showed, significantly higher levels of P‐selectin/CD40L expression and intra‐platelet content of TGF‐β1 were detected shortly after transfusion (2‐h post‐surgery). At the same time, PAC‐1 binding and NLR increased in patients with platelet transfusion compared to those without transfusion, with a delay of 24 h after surgery. One week later, while there was no difference between the two groups in terms of PAC‐1 binding and TGF‐β1 content, where they returned to similar levels before surgery, the expression of P‐selectin and CD40L was higher in patients without platelet transfusion compared to patients who had transfusion. In addition, the levels of these pro‐inflammatory molecules in patients without platelet transfusion were even higher than in their pre‐surgery condition. However, unlike the shorter postoperative period, this increase in platelet inflammatory markers was not associated with increased levels of NLR after a week.

To the best of our knowledge, no data reveals the effects of platelet transfusion on platelet function in post‐CABG patients. However, several lines of evidence deeply examined the post‐CABG state of platelet function in patients without considering the status of platelet transfusion. As we also showed in our recently published paper, these studies indicated significant increases in the levels of inflammatory cytokines/chemokines that can affect platelet functional activity immediately after surgery, where they showed significant increments in platelet P‐selectin and platelet‐leukocyte aggregates (PLA). For instance, Untch et al.^31^ demonstrate that P‐selectin and PLA levels significantly increased in patients 2 h after CABG. Vallely et al.^32^ also spotlighted that platelet activation (P‐selectin expression/release) and platelet injury are well‐recognized phenomena after cardiac surgery with CPB. In another study, as shown by Kondo and colleagues, the expression of P‐selectin increased within 5 min after the start of the CPB, where it peaked until 2 h after the bypass, which was about 1.4 times the level before the bypass.^33^

Other studies have also shown that in parallel with the increase in P‐selectin expression after CPB, the formation of PLA is also increased, evidence supporting the occurrence of a functional pro‐inflammatory phenotype of platelets. In this regard, Serrano et al.^34^ suggested that in stable CAD patients undergoing CABG, the activation of leukocytes and platelets and their interactions (PLAs) are essential signs of inflammatory processes after surgery. Rynder and colleagues also demonstrated a gradual increase in platelet‐monocyte conjugates throughout the CPB, which was subsequently accompanied by a decreased level after bypass. They also showed that platelet–neutrophil conjugates peaked 10 min after the start of CPB, while they decreased more slowly in comparison.^35^ In another study that highlighted the post‐CABG pro‐inflammatory function, Ivert et al.^19^ found that circulating PLAs of all subtypes (platelet‐conjugated monocytes, neutrophils and lymphocytes) were elevated 1 month after surgery, where agonist‐stimulated PLA formation ex vivo was also enhanced at this time point. This was consistent with their previous studies in which they showed that CABG can induce early (within 1 week) activation of circulating platelets and leukocytes, increased circulating PLA numbers and significant activation of inflammatory responses. They concluded that the induction of platelet response and the increase of PLA are caused by a long‐term inflammatory process that sometimes does not return to normal even after 3 months.^18^, ^19^, ^36^ Correspondingly, some of these studies have also shown increases in platelet‐released levels of inflammatory/immunomodulatory markers, such as soluble P‐selectin and TGF‐β1 during CABG.^17^, ^31^, ^37^, ^38^ One of these studies is that of Komai and Haworth,^39^ who showed a parallel increase in soluble P‐selectin and plasma β‐thromboglobulin (both found in platelet α‐granules) after CABG. At the same time, Sablotzki et al. also showed that the level of soluble TGF‐β1 also increased 20 min after the start of CABG, which reached its highest level by the end of surgery.^40^ Moreover, Denizot et al. also showed that the levels of soluble TGF‐β1 increased significantly immediately after the CABG compared to the pre‐surgery values and remained high until the patient was removed from the pump and returned to the baseline level 6 h after the surgery.^41^ However, the longer‐term follow‐up of these variables in time intervals of more than a week has often been accompanied by a decrease in their values to pre‐surgery levels and even lower rates, which was usually considered to be indicative of a reduction in the post‐surgery inflammatory state or the success of the CABG operation.^18^ However, none of these studies specifically clarified the state of platelet transfusion in these patients. In contrast, no separate or comparative studies have been presented so far that deal with the effect of platelet transfusion on the evidence above.

**FIGURE 6:**
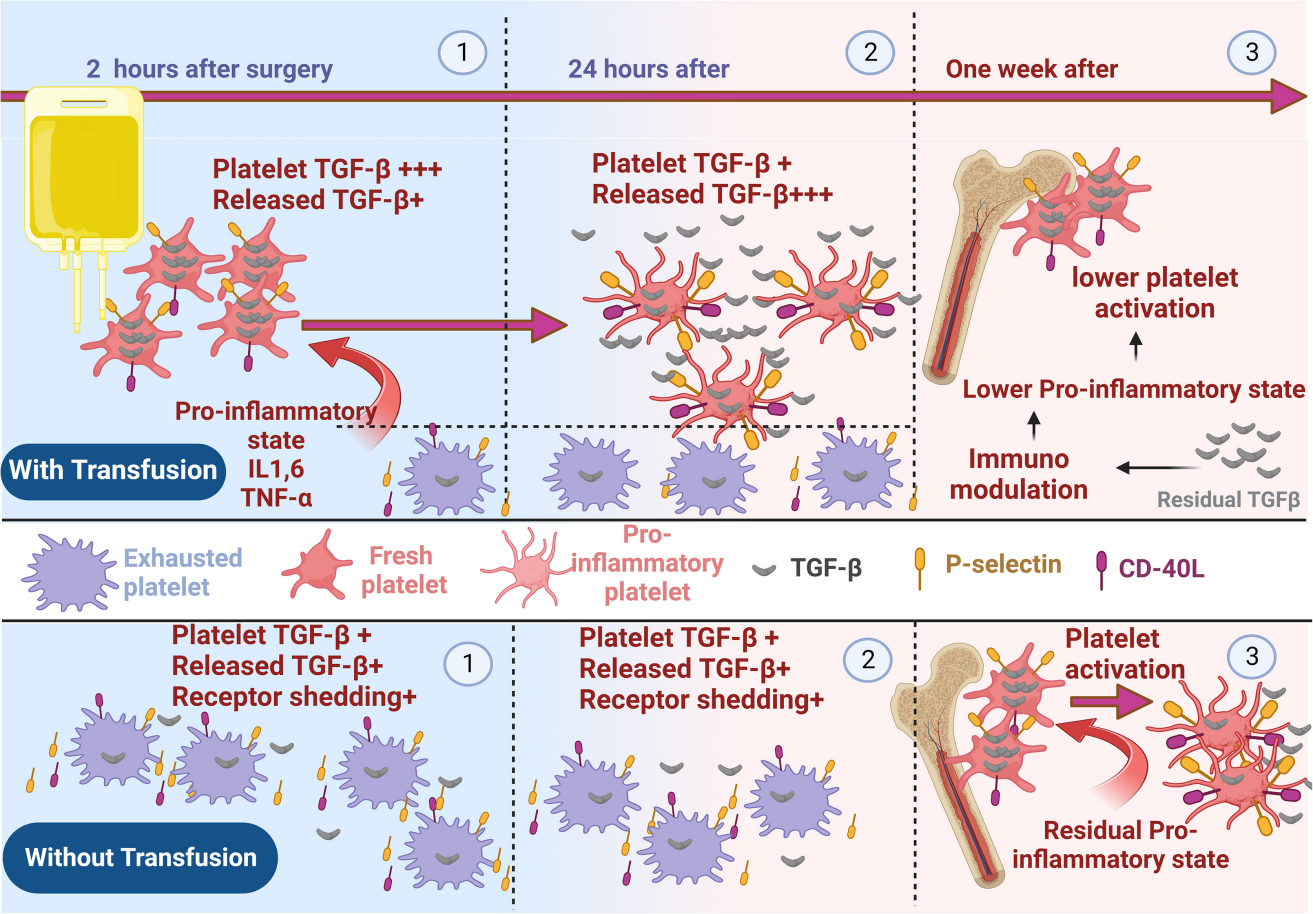
The modulatory effects of platelet transfusion on the pro‐inflammatory state of platelets in CABG patients. Bottom: (1) and (2) Patients undergoing CABG experience a systemic inflammatory state mainly due to surgical stress, which together with possible artificial effects of pumps can lead to platelet activation. This activation is characterized by the significant expression of pro‐inflammatory markers P‐selectin and CD40L, as well as the release of TGF‐β1 shortly after surgery. However, 2–24 h after surgery, platelets lose their expressed pro‐inflammatory molecules and their TGF‐β1 content, down to the lowest level, rendering them to an exhausted state. (3) One week after surgery, newly produced platelets by the bone marrow are also partially activated in the face of residual inflammatory condition. Top: (1) 2 h after surgery and post‐platelet transfusion, the presence of fresh platelets at lowest activity state elevates platelet TGF‐β1 content while the expression of P‐selectin and CD40L also increase compared to non‐transfused patients. The increased expression of P‐sel/CD40L here could be due to the exposure of fresh platelets with good activation potential to the post‐surgical inflammatory milieu (2) however, 24 h after surgery exposing to inflammatory milieu, platelet TGF‐β1 content and pro‐inflammatory expression are reduced due to granule release and shedding events respectively. (3) As transfused platelets released their TGF‐β1 contents, a week after surgery, their immunomodulatory effects may settle down inflammatory conditions and hinder the activation of freshly generated platelets by the bone marrow. This may be a potential explanation for the decreased expression of P‐selectin and CD40L in patients with platelet transfusion compared to those who did not receive therapeutic platelets.

In this study, P‐selectin and CD40L expression in patients who underwent surgery with or without platelet transfusion revealed a similar pattern of increased expression immediately after surgery, followed by a decrease to preoperative levels within 24 h. However, in transfused patients, the levels of P‐selectin and CD40L expression were significantly higher than those without platelet transfusion 2 h after CABG. While the increases of these activation markers of platelets immediately after surgery and their subsequent attenuations followed by the repression mechanisms or platelet exhaustion can be attributed to the post‐surgery inflammatory flux within a surgery‐dependent stressful condition,^42^ higher levels of these markers after platelet transfusion compared to non‐transfused patients may need further consideration. As a possible mechanism, whereas post‐surgery exhausted platelets are in metabolic distress and unable to replenish their functional expression due to the irreversible release of their internal sources, the subjection of fresh platelets during transfusion and their interaction with inflammatory milieu leads to a renewed increase in the expression of platelet activity markers (Figure 6). However, in the face of persistent stimulating conditions, these platelets also lose their surface molecules due to ectodomain shedding and irreversible release of their internal sources, which also converts them to an exhausted phenotype up to 24 h after surgery.^43^

Meanwhile, more interestingly, 1 week after surgery, a new expression pattern of platelet markers appeared, in which the expression levels of these molecules were higher in patients without a history of platelet transfusion than in transfused patients. Now, whether with the same scenario in patients who have not been transfused, the exposure of released newly produced platelets by the bone marrow to a lasting inflammatory condition could be the reason for this observation is an exciting point of discussion, especially that in a previous research Li et al. also showed a similar pattern albeit without considering platelet transfusion.^18^ However, on the other hand, the observation of platelets with lower levels of these activation markers in patients with a history of platelet transfusion in this study was another interesting finding, and whether this observation might be due to the immunomodulatory effects of platelet transfusion in controlling the underlying inflammatory condition and then platelet activation^44^, ^45^ was an important issue that required further investigation.

For this reason, the total (intra‐platelet) content of TGF‐β1 was also evaluated as an essential marker, and its reduction during platelet activation highlights the immunomodulatory state^46^ in the patients before and after surgery. In this regard, a consistent decrease in the intra‐platelet content of TGF‐β1 in CABG patients, in parallel with some previous studies that showed increasing levels of soluble TGF‐β1 after surgery,^37^, ^40^, ^41^ points to the potential role of platelet in post‐CABG immunomodulation. Here, the observation of a sharp increase in the intra‐platelet TGF‐β1 content after platelet transfusion due to supplementation of patients' blood with fresh platelets, followed by its rapid decrease until 1 week later, may be the reason for the lower platelet activity/pro‐inflammatory function during this period compared to patients without transfusion. This indirect evidence suggests how TGF‐β1‐mediated immunomodulation can temper down post‐CABG inflammatory state, especially in platelet‐transfused patients. However, further studies comparing the levels of inflammatory cytokines in patients who received platelets with those who did not are recommended, as such studies may provide more mechanistic evidence for the reduction in platelet functional activity observed after platelet transfusion.

On the other hand, since the biological response of each cell to progressive inflammatory stages involves a multifactorial transcriptional network that is changing as inflammation progresses,^47^ in addition to single‐factor approaches (such as measuring TGF‐β1), global and network‐based ones can also help to better understand the effects of transfused platelets on the systemic inflammatory state (especially in a time scale after platelet transfusion).

In addition to the granule‐released markers potentially involved in platelet pro‐inflammatory/immunomodulatory function, monitoring the pro‐aggregatory status of platelets provided by integrin activation was also of interest for this study. Regardless of the platelet transfusion in patients undergoing surgery, studies have yet to show evidence of an increase in pro‐aggregatory conditions after surgery. This observation may be due to the successful control of intraoperative hemostatic function.^48^, ^49^ Notably, as presented here, while non‐transfused patients did not show significant changes in PAC‐1 binding via MFI analysis, surprisingly, 24 h after the platelet transfusion, the patients showed an increase in PAC‐1 binding that was significantly higher than those who had not been transfused. This finding is consistent with the results obtained for P‐selectin and CD40L expression, with the difference that the levels of PAC‐1 binding occurred 24 h later than the increased expression of pro‐inflammatory molecules. For this study, later increases in PAC‐1 binding than P selectin/CD40L expression were expected as compared to granule release; the full integrin activation requires accumulative inside‐out signals with a significant Ca2^+^ influx.^50^ It is also noteworthy that, according to our parallel studies, stored platelets of the same age as pre‐transfusion platelets had lower expressions of P‐selectin, CD40L and PAC‐1 binding (data not shown). These observations confirm the effects of the post‐surgery milieu on increasing the activation of transfused platelets.

## 5 CONCLUSION

The study presented here showed that the administration of platelet transfusion in individuals undergoing CABG has resulted in immediate, midterm and delayed changes in platelet functional phenotypes. While the significant rise in the pro‐inflammatory state of platelets is considered an immediate effect of platelet transfusion in these patients, the pro‐aggregatory status, which occurs later at 24 h after surgery, may require further clinical attention. However, a week after surgery, transfused patients showed a lower pro‐inflammatory state of platelets, which might be due to the higher levels of TGF‐β1 released from transfused platelets that modulate the effects of later inflammatory status on the newly generated platelets by the bone marrow. Given our previous studies on freshly stored platelets (with the storage times used in this study) obtained from volunteers donors, these products showed almost low levels of pro‐inflammatory and proaggregatory states (regarding P‐selectin and PAC‐1 binding) with the acceptable pro‐adhesive function (in terms of the expression/shedding levels of adhesion receptors and platelet adhesive capacity to fibrinogen and collagen matrices) as wells as a an acceptable immunomodulatory potential (given high levels of intra‐platelets TGF‐β1).^30^, ^51^, ^52^ This means that transfused platelets provided a good source of functional platelets with acceptable levels of platelets mediators including TGF‐β1, which could account for the increase of this immunomodulatory mediator under activating state after transfusion in patients. However, regardless of the fact that patients' exhausted platelets may not respond well to post‐surgery inflammatory stimuli, platelet activation after transfusion might be contributed in part by the recipient's own platelets rather than transfused platelets. Similarly, the relative contribution of these two pools of platelets to the net TGF‐β1 production has also not been examined in this study. These issues, even if it is not possible to handle them on the bench, are still considered as limitations of this study.

Notably, attributing the lower pro‐inflammatory state of platelets to increased intraplatelet TGF‐β1 content 1 week after blood transfusion and its immunomodulatory effects on newly generated platelets by the bone marrow still needs further investigation, first to demonstrate the inhibitory effects of TGF‐β1 on the platelet inflammatory profile in validated in vitro/in vivo models and then to show the relationship between platelet‐released TGF‐β1 levels and immature platelet fraction/count (IPF/C) at time intervals after surgery,^53^ both in patients with and without platelet transfusions. In addition, regardless of the possible direct effects of TGFβ1 on platelets, its pleiotropic interplay with the inflammatory state may provide a new horizon for future research, in particular, where studies showed the anti‐inflammatory effect of TGFβ1.^54^, ^55^ This effect especially reduces systemic inflammatory response syndrome (SIRS),^56^ a clinical condition that usually occurs following CABG^57^, ^58^, ^59^ and can indirectly cause platelet activation.^60^, ^61^ More interestingly, in a recent study, Wang et al.^62^, ^63^ demonstrated how platelet TGF‐β leads to restoration of the immune misbalance in ITP via the reprograming myeloid‐derived suppressor cell (MDSC) function, a process that increases platelet recovery in these patients. However, regardless of the unique effect of TGFβ1, it should be noted that other inflammatory signatures and expression of activation markers are tightly associated with the stemness/differentiation juncture on the haematopoietic axis,^64^ an important reciprocal crosstalk that may differentially affect the nature of platelet production and release from the bone marrow in transfused and non‐transfused patients.

## AUTHOR CONTRIBUTIONS

**Javad Ahmadi:** Data curation (equal); formal analysis (equal); investigation (equal); software (equal); writing – original draft (equal). **Ehteramolsadat Hosseini:** Conceptualization (equal); data curation (equal); formal analysis (equal); investigation (equal); methodology (equal); software (equal); validation (equal); visualization (equal); writing – original draft (equal). **Faranak Kargar:** Conceptualization (supporting); investigation (supporting); methodology (supporting). **Mahtab Maghsudlu:** Conceptualization (supporting); formal analysis (supporting). **Mehran Ghasemzadeh:** Conceptualization (equal); data curation (supporting); formal analysis (lead); funding acquisition (lead); investigation (lead); methodology (equal); project administration (lead); resources (lead); software (equal); supervision (lead); validation (lead); visualization (lead); writing – review and editing (lead).

## FUNDING INFORMATION

This work is a part of the project of Dr. Hosseini and Dr. Ghasemzadeh, which was approved and supported by the High Institute for Research and Education in Transfusion Medicine.

## CONFLICT OF INTEREST STATEMENT

The authors declare that they have no competing interests.

## Supporting information


Figure S1.

## Data Availability

The corresponding author can make available some datasets upon reasonable request.
