# Supplementary material for: Platelet transfusion enhances pro‐aggregatory status shortly after coronary artery bypass grafting (CABG while modulating platelet pro‐inflammatory state 1‐week post‐surgery
Source: J Cell Mol Med. 2024 Aug 9;28(15):e18573. doi: 10.1111/jcmm.18573 (PMC11315096; doi:10.1111/jcmm.18573)
Supplement: Supplementary file 1 — Figure S1. [file JCMM-28-e18573-s001.docx]

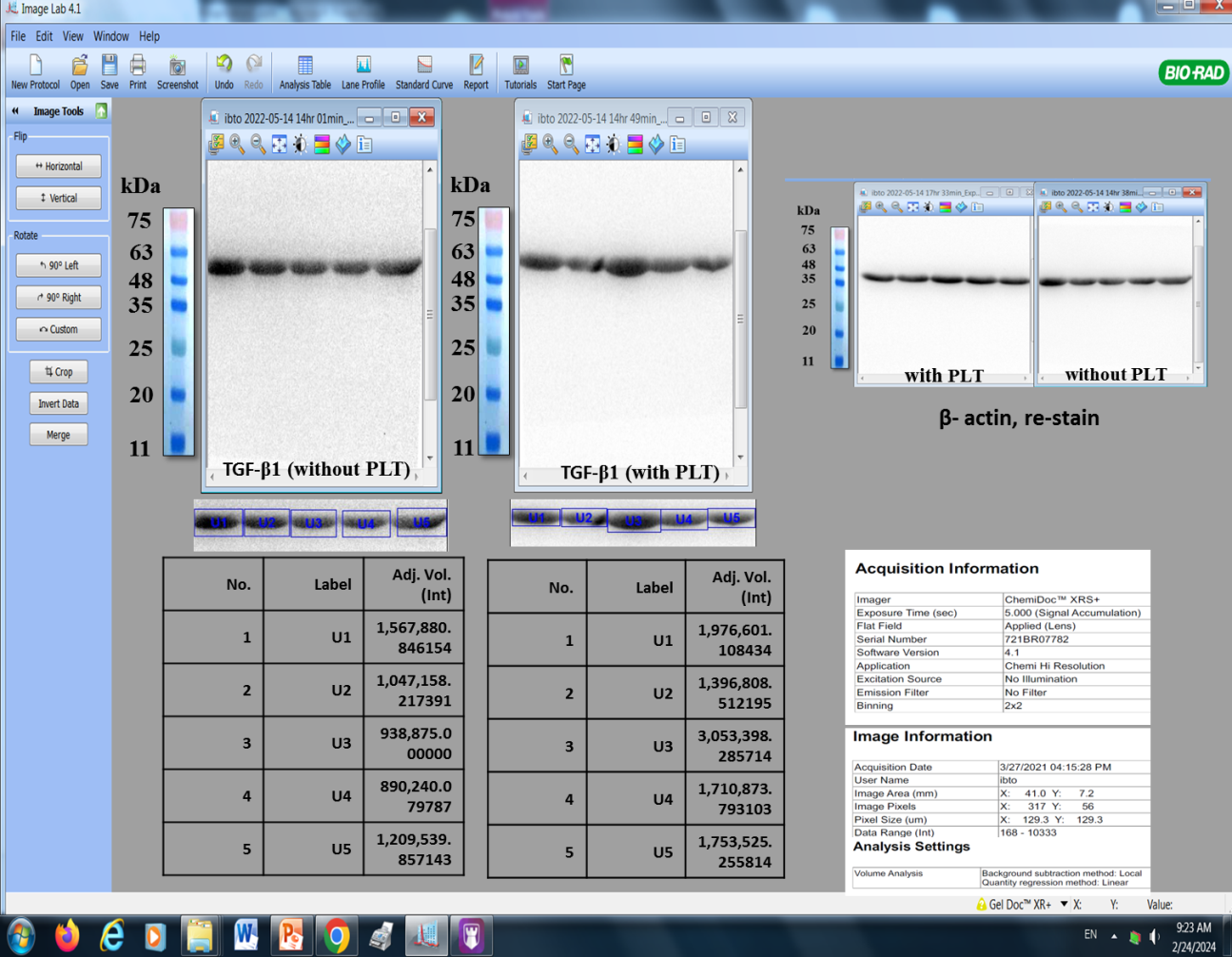


**Supplementary Figure1** demonstrates the original blots of figure 4. Here followed by SDS-PAGE electrophoresis PVDF membrane was subjected to western blotting where the TGF-β1 protein was immunoblotted sequentially in the presence of TGF-β1-specific monoclonal antibody, a secondary antibody coupled to HRP, and the enhanced chemiluminescence reagent as already mentioned. Finally, the bands were visualized and analyzed by the ChemiDoc XRS+ equipment and Imaging Lab software (Bio-Rad Laboratories, Inc. USA). The membrane was then washed vigorously (with TBST +0.1% Tween 20) and re-blocked with 5% skimmed milk in PBS and subjected to 2^nd^ phase of antibody staining against β actin, as a hose-keeping protein. New emerging bands were visualized and analyzed by ChemiDoc XRS+ as mentioned above.
